# Relationship of Sternal Foramina to Vital Structures of the Chest: A Computed Tomographic Study

**DOI:** 10.1155/2013/780193

**Published:** 2013-10-10

**Authors:** J. Gossner

**Affiliations:** Evangelisches Krankenhaus Göttingen-Weende, Department of Clinical Radiology, An der Lutter 24, 37074 Göttingen, Germany

## Abstract

Sternal foramina are a well-known variant anatomy of the sternum and carry the risk of life-threatening complications like pneumothorax or even pericardial/cardial punction during sternal biopsy or acupuncture. There have been numerous studies numerous studies examinimg prevalence of sternal foramina, but the study of the exact anatomical relationship to intrathoracic structures has received little attention. In a retrospective study of 15 patients with sternal foramina, the topographical anatomy in respect to vital chest organs was examined. In most patients, the directly adjacent structure was the lung (53.3%) or mediastinal fat (33.3%). Only in three patients, the heart was located directly adjacent to a sternal foramen (20%). Theoretically, if the needle is inserted deep enough it will at some point perforate the pericardium in all examined patients. There was no correlation between the patient habitus (i.e., thickness of the subcutaneous fat) and the distance to a vital organ. In this sample, pericardial punction would have not occured if the needle is not inserted deeper than 2.5 cm. Given the preliminary nature of the data, general conclusions of a safe threshold for needle depth should be made with caution. To minimize the risk of hazardous complications, especially with sternal biopsy, preprocedural screening or image guidance is advocated.

## 1. Introduction

**Figure 1:**
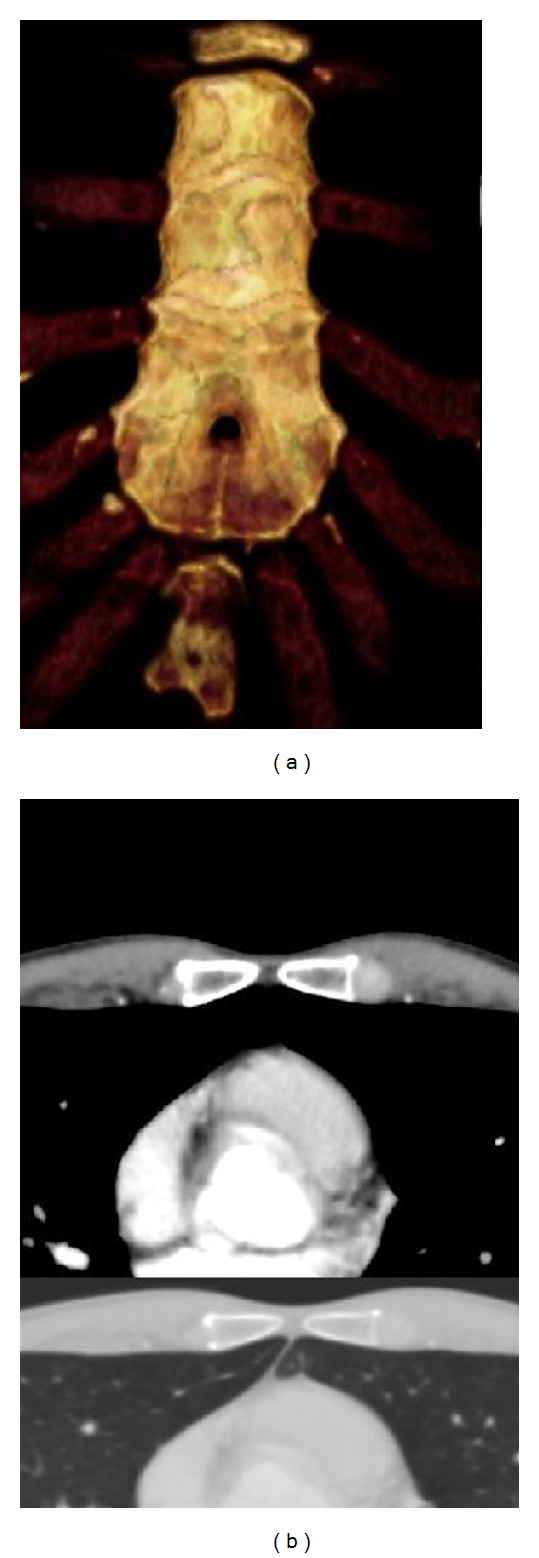
Computed tomographic images of a typical sternal foramen. The axial slices (b) are showing the close contact to vital structures (lung and heart). There is a potential risk of life-threatening complications (pneumothorax/cardiac tamponade), if a needle is inserted unintentionally through this foramen.

Sternal foramina are a well-known variant anatomy of the sternum with reported prevalence between 4.3 and 6.7% [1, 2]. These foramina are developmental anomalies because of incomplete fusion of ossification centers; most often they are located at the caudal parts of the corpus sterni [3, 4]. In most cases, these foramina are isolated developmental anomalies [4]. Because of the close contact to the mediastinum and therefore vital structures of the chest, there is a risk of life-threatening complications like pneumothorax or even pericardial/cardiac puncture during sternal biopsy or acupuncture (Figure 1) [5, 6].

There have been numerous studies examining the prevalence of sternal foramina. In contrast, the study of the topographical anatomy in respect to intrathoracic structures has received little attention. Only one morphological study reported that all needles which were inserted through a sternal foramen will eventually perforate the heart [7]. To avoid serious complications more knowledge of the topographical relationship to vital structures of the chest would be of great interest for the clinician performing sternal biopsies or acupuncture. Using computed tomographic (CT) scans of the chest, this study examine the exact topographical relationship between sternal foramina and intrathoracic organs.

## 2. Material and Methods

This retrospective study of chest CT scans was performed in accordance with the statute of the ethics committee of the affiliated University of Göttingen. All CT scans of the chest performed during a 5-month period (mid-February to mid-July) at the Department of Clinical Radiology of Weende Hospital were retrospectively reviewed for the existence of sternal foramen. Sixteen patients with sternal foramina were found in the examined 352 chest CT scans. Patients were imaged for a wide range of indications including evaluation of lung disease (for example, emphysema and fibrosis), suspected pulmonary embolism, and staging of malignant tumors. In one patient with a sternal foramen, mediastinal anatomy was grossly altered due to chronic infection of the left lung with volume loss and consecutive mediastinal shift. This patient was excluded from the analysis. Finally, 15 sternal foramina were analyzed. The sample consisted of 9 men and 6 women with a mean age of 69.5 years (range 49–90 years). All patients were examined using the same parameters (120 kV, 80 mAs, 1 mm slice thickness, and automatic exposure control) on a 16-slice scanner (Activision, Toshiba Medical Systems, Tokyo, Japan). Scans were acquired during breath hold in deep inspiration. The primary data sets were examined using the departmental PACS (synedra view, Innsbruck, Austria). All scans were reviewed by the author (consultant radiologist). Topographical relationships to intrathoracic organs were analyzed (adjacent mediastinal fat, lung, or heart) and the following measurements were made: distance from the skin to the sternal foramen (i.e., thickness of the subcutaneous fat), width of the foramen, distance from the foramen to a vital structure (lung = pneumothorax and pericard = pericardial tamponade), and, if applicable, distance from the skin to the pericard. A correlation between the thickness of subcutaneous fat and distance to a vital structure was performed. 

## 3. Results 

**Figure 2:**
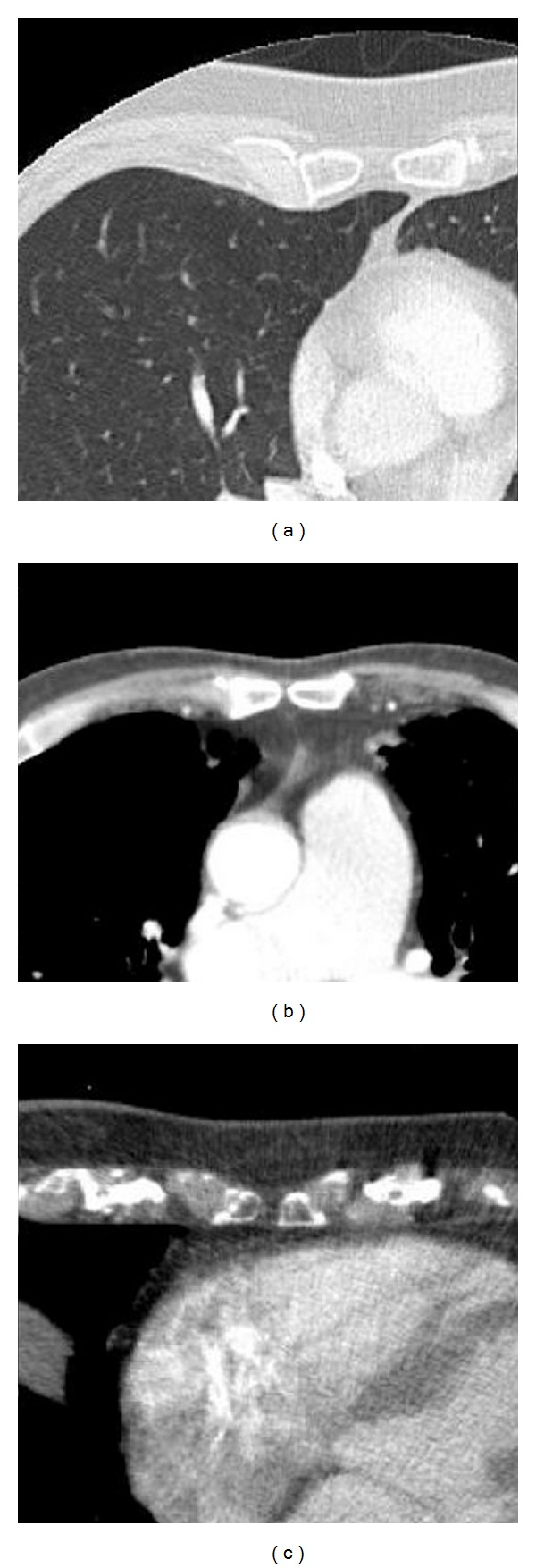
Topographical anatomy in three different patients. Most commonly there was lung directly adjacent to the sternal foramen (a), less commonly there was adjacent mediastinal fat (b), and only in 3 patients, the pericard/heart was directly adjacent to a sternal foramen (c).

All found sternal foramina were located in the typical position at the caudal parts of the corpus sterni. Prevalence of sternal foramina in this sample was 4.5%. The mean width of sternal foramina was 3.3 mm. In most patients, the directly adjacent structure was the lung (53.3%; 8 of 15) or mediastinal fat (33.3%, 5 of 15). Only in three patients the pericard/heart was located directly adjacent to a sternal foramen (20%, 3 of 15) (Figure 2). 

In patients with directly adjacent lung, these were located directly behind the foramina, the same was found for patients with directly adjacents pericard/heart. In all patient, the pericard would be perforated if a needle was inserted deep enough, the mean distance from the skin to the pericard was 4.96 cm (range 3–9.1 cm). That is, in this sample no pericardial puncture would have occurred if the needle was inserted less than 3 cm. In all but one patient the heart could be reached, in the remaining patient perforation of the large thoracic vessels (ascending aorta or pulmonary trunk) would have occurred with excessive deep needling. There was no correlation between the thickness of the subcutaneous fat and the distance to a vital structure. The whole data is presented in Table 1. 

## 4. Discussion

Different anatomical variations can be found at the human sternum. Because of the risk of incidental fatal cardiac tamponade with acupuncture or sternal biopsy complete sternal foramina have received most attention [1, 2, 4–7]. Like described previously a detailed description of the topographical anatomy of sternal foramina to vital chest organs has not been performed before. There are two main organs with the potential for life-threatening complications. Firstly, puncture of the lung may cause pneumothorax, and secondly, and even more dangerously, punction of the pericard may result in pericardial tamponade. In their anatomical study, Babinski et al. inserted needles through the found sternal foramina in 16 cadaveric specimen and found that eventually every needle will puncture the heart [7]. They did not give a measurement of the distance from the foramen to the heart. Their finding was confirmed in this study, but the depth from skin to the pericard varied considerably from 3 to 9.1 cm. That is, taking a safety margin into account, if the needle had been inserted not deeper than 2.5 cm, pericardial puncture would have not occurred in this sample. In most patients, the direct adjacent structure was the lung or mediastinal fat, and only in 3 patients the pericard/heart was located directly dorsal to a sternal foramen. Interestingly, there was no correlation between the depth of the subcutaneous fat and the distance to a vital structure. In conclusion patient habitus is not giving any clue on mediastinal anatomy and therefore the risk for dangerous puncture. From a clinical point of view, this high variability has to be considered with needle placement in acupuncture or sternal biopsy. Generally, needle placement should be undertaken carefully and if it appears deeper than the adjacent sternal body suspicion of a sternal foramen should be made. As sternal foramina can be seen on ultrasound, a screening of potential dangerous variant anatomy before sternal puncture is easily possible [8]. Bone marrow biopsy should be performed at the iliac crest and sternal biopsy should be avoided. If biopsy of the sternum is needed, it can be safely performed under CT guidance. The prevalence of sternal foramina in this study (4.5%) is in accordance to most other studies reporting prevalence between 4.3 and 6.7% [1, 2]. Only Babinski et al. reported a higher prevalence of 16.6%. Because of the similar prevalence of most studies, our sample seems to be comparable to other examined populations. But given the found anatomical variability in the presented relatively small sample, the presented data is preliminary and needs to be replicated in other populations before general conclusions can be drawn. Especially conclusions about a safe threshold for needle depth should be made with caution. 

## 5. Conclusion

Sternal foramina are a quite frequent variant anatomy with the risk of serious complications like pneumothorax or pericard tamponade during sternal biopsy or acupuncture. The topographical anatomy in the studied patients was highly variable, and especially that there was no correlation between patient habitus and the distance to a vital organ. In more than 50% of patients, the direct adjacent structure dorsal to sternal foramen was the lung with the risk of pneumothorax. In this sample, pericardial punction would not have occurred if the needle had not been inserted deeper than 2.5 cm. Given the preliminary nature of the data, general conclusions of a safe threshold for needle depth should be made with caution. To minimize the risk of hazardous complications, especially with sternal biopsy, preprocedural screening or image guidance is advocated. 

## Figures and Tables

**Table 1:** Complete data of the 15 examined patients.

Patient	Age (years)	Structure directly adjacent to the foramen	Width of foramen (mm)	Thickness of subcutaneous fat (cm)	Distance from foramen to a vital structure	Distance from the skin to the pericardium (cm)
1	69	Lung	5	2.2	0	6
2	51	Lung	0.5	1.8	0	5.3
3	63	Lung	1	1	0	5.1
4	90	Mediastinal fat	3.8	2.1	4.7	7.4
5	55	Lung	2	1.1	0	3.7
6	58	Mediastinal Fat	2.8	0.4	1.7	3.1
7	83	Pericard/heart	4.3	1.7	1	3.9
8	69	Lung	2.7	0.8	0	4.5
9	88	Pericard/heart	5	1.9	0	2.9
10	85	Pericard/heart	1.4	1.2	0.5	3
11	72	Lung	3.4	1.5	0	9.1
12	57	Mediastinal fat	2.1	0.8	2.7	4.2
13	83	Mediastinal fat	5	1.3	2.8	5.2
14	70	Lung	1	2.1	0	5.5
15	49	Lung	10	1.9	0	5.6

Mean	69.5		3.3			4.96
